# Ryan White HIV/AIDS Part B and AIDS Drug Assistance Programs during COVID-19: safety net public health programs’ challenges and innovations

**DOI:** 10.3389/fpubh.2023.1172009

**Published:** 2023-07-31

**Authors:** Kathleen A. McManus, Andrew M. Strumpf, Amy Killelea, Tim Horn, Amber Steen, Zixiao An, Elizabeth Schurman, Auntré Hamp, Jessica Keim-Malpass

**Affiliations:** ^1^Division of Infectious Diseases and International Health, University of Virginia School of Medicine, Charlottesville, VA, United States; ^2^Killelea Consulting, Arlington, VA, United States; ^3^NASTAD, Washington, DC, United States; ^4^Center for Global Health Equity, University of Virginia, Charlottesville, VA, United States; ^5^Department of Pediatrics, University of Virginia School of Medicine, Charlottesville, VA, United States

**Keywords:** AIDS Drug Assistance Program, HIV, COVID-19, Ryan White HIV/AIDS Program, public health practice

## Abstract

**Introduction:**

We characterized the challenges and innovations of states’ Ryan White HIV/AIDS Program (RWHAP) Part B programs, including AIDS Drug Assistance Programs (ADAPs), during the COVID-19 pandemic. In the United States, these are important safety net programs for HIV healthcare, providing essential medical and support services, and medications, to people with HIV with low incomes who are uninsured/underinsured.

**Methods:**

Data were collected via the 2021-2022 NASTAD National RWHAP Part B and ADAP Monitoring Project Report, a cross-sectional survey of state, district, and territorial programs through a mixed method study design. For quantitative data, we used descriptive statistics. Qualitative responses were coded and analyzed using content analysis.

**Results:**

Forty-seven RWHAP Part B and ADAPs responded (92% response rate). The majority of respondents reported that maintaining client eligibility (78%) and working remotely (70%) were the most challenging aspects of the pandemic, particularly in regards to implementing new telehealth and e-certification platforms. In response to COVID-19, programs introduced enrollment “grace periods” (19%), bolstered client outreach (11%), allowed more than a 30 day supply of medications (79%), and supported medication home delivery for clients (80%).

**Discussion:**

Despite the challenges of the COVID-19 pandemic, RWHAP Part B and ADAPs implemented several operational innovations in order to continue providing essential medicines and services. Other public health programs may adopt similar innovations, including digital innovations, for greater public health benefit. Future studies should assess the retention of policy innovations over time, their impact on the individual client level satisfaction or health outcomes, and what factors may improve the acceptability of telehealth and e-certification platforms.

## 1. Introduction

In the United States (US), Ryan White HIV/AIDS Program (RWHAP) state Part B programs and state AIDS Drug Assistance Programs (ADAPs) are key pillars of the HIV healthcare delivery safety net. State RWHAP Part B programs support core medical and support services in all 50 states, the District of Columbia, and US territories (1). RWHAP Part B also include ADAPs, which provide free medications, including antiretroviral therapy, or subsidized insurance plan coverage to people with HIV with low incomes who are uninsured/underinsured (2). In 2020, the COVID-19 pandemic upended the economy and led to record unemployment (3). The demand on income eligibility-based safety net programs, such as RWHAPs and ADAPs, may surge when economic disruptions occur (4, 5). Thus far, HIV and COVID-19 have been co-located in geographic areas with greater poverty and unemployment, highlighting how adverse social determinants can amplify disease burden and attenuate public health responses in these communities (6–9). Because RWHAP Part B and ADAPs are a “payer of last resort,” they work in tandem with other health coverage programs (10). Therefore, the federal and state expansion of broader safety net programs in response to COVID-19 directly impacted how Part B programs and ADAPs could respond. For example, the federal requirement that state Medicaid programs provide continuous coverage during the Public Health Emergency (PHE) (11) undoubtedly sustained medication access for many people with HIV and likely led to less of a surge in need for ADAP support.

Recently, the Kaiser Family Foundation surveyed directly-funded RWHAP medical provider grantees during the COVID-19 pandemic and reported on additional aspects of the impact of COVID-19 on HIV care (12, 13). They found that while many RWHAP medical provider grantees had operating challenges, grantees adjusted in many ways including by using telehealth and offering COVID-19 testing. Despite this work, the experience of state RWHAP Part B programs and ADAPs during COVID-19 has not been described in the published literature. ADAPs are in a unique position as a safety net public health program based at state health departments. Additionally, while ADAPs are federally mandated, they are funded by a combination of federal and state funds, and most implementation decisions are made at the state level (5, 14). RWHAP Part B program implementation also has a lot of flexibility at the state level. Our objective was to explore how RWHAP Part B and ADAPs responded to the COVID-19 pandemic, what challenges they faced, and what innovations were developed to overcome barriers and maintain service delivery.

## 2. Materials and methods

### 2.1. Data collection

Data were collected as part of the 2021–2022 National Alliance of State & Territorial AIDS Directors (NASTAD) National RWHAP Part B and ADAP Monitoring Project Report, an annual cross-sectional survey of state and territorial programs (15). The survey reports on utilization, expenditures, and client outcomes. Data on programs’ practices during the COVID-19 pandemic to-date were collected between May and July 2021. This study was reviewed by the University of Virginia Institutional Review Board and was determined to be non-human subject research.

To understand the impact of COVID-19, questions were added to the 2021–2022 Monitoring Project survey. They included: 7 Likert-style questions assessing the impact of COVID-19-related challenges for ADAPs, 3 Likert-style questions regarding specific innovations ADAPs implemented to address the challenges, and 3 open-ended, text-entry based questions for both programs to detail challenges and innovations that affected their specific programs (Supplementary material 1). State program leaders were asked to complete the questions. NASTAD and University of Virginia staff assessed data quality to ensure response accuracy.

### 2.2. Quantitative data analysis

We calculated overall response rates for each question and reported at the national and regional level. Regions were defined according to the US Census Bureau: Northeast, Midwest, West, and South (16). At the regional level, we evaluated differences in categorical responses by calculating proportions and applied Fischer’s exact test. Data were analyzed using R Studio (17). *P* values <0.05 are reported in the text.

### 2.3. Qualitative data analysis

Text responses were transcribed verbatim. We used both an inductive and directed coding approach to guide the qualitative analysis using content analysis (18). An initial codebook was developed using an open coding approach and constant comparison to describe phenomena of interest described from the program’s experience. We assessed the richness and quality of the data concurrently throughout the iterative development and refinement of the codebook. We maintained field notes to document the iterations and refinements. The codebook was then applied in a directed approach independently by two reviewers. The initial applications were compared by calculating inter-rater agreement (Krippendorff’s alpha) (19). Codes and descriptions applied inconsistently by reviewers were revised and resolved by consensus.

Due to the nature of the interview questions prompting discussion of difficulties and allowances made during the pandemic, codes were grouped conceptually into two topics: challenges and innovations. Each topic contained codes, related sub-codes, code application frequency, code presence frequency, and exemplar quote(s). To maintain rigor, decisions regarding the analysis (re-parenting or merging codes) were open to all members of the study team (20). All aspects of the codebook development and application were managed using Dedoose (21).

Quantitative and qualitative results are described together topically in results. We created a situational map that depicts codes present in >5% of responses. Based on responses and reviewer interpretation, interconnected sub-codes are represented using lines. Arrows were added when reviewers interpreted a relational connection.

## 3. Results

**Figure 1:**
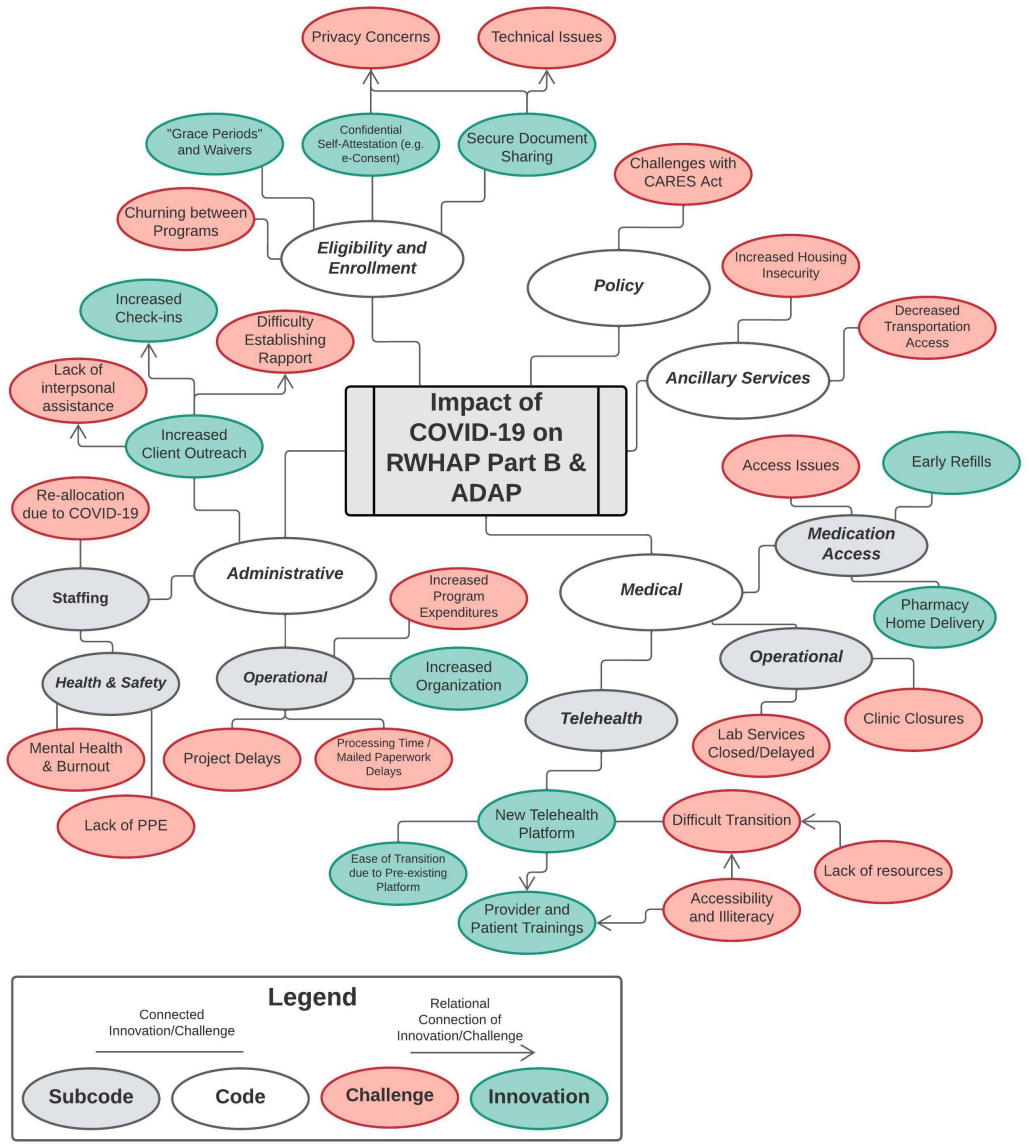
Situational map of codes and sub-codes, describing the challenges of COVID-19 and subsequent innovations implemented by ryan white HIV/AIDS program Part B and AIDS drug assistance programs.

Forty-seven programs responded to the COVID-19 Likert-style questions as part of the Monitoring Project survey yielding a national response rate of 92%. Regionally, the sample consisted of 11 Midwest (92%, response rate), 9 Northeast (100%), 15 South (88%), and 12 West (92%) jurisdictions. Quantitative findings are in Tables 1, 2. Forty-five programs answered the open-ended questions yielding a national response rate of 88%. The initial codebook applications achieved a Krippendorff’s alpha of 0.82, indicative of strong agreement. For the qualitative analysis, codes, presence, frequency, and representative quotes are in Supplementary Table 1 (challenges) and Supplementary Table 2 (innovations). In addition, codes and sub-codes are depicted in a situational map (Figure 1). The topics of challenges and innovations are described in five codes: (1) Eligibility and Enrollment, (2) Administrative, (3) Medical, (4) Ancillary Services, and (5) Policy.

**Table 1:** Challenges during first year of the COVID-19 pandemic for AIDS drug assistance programs, overall and by region, 2020.

Challenges	Total (*n* = 47)		Region								
			Midwest (*n* = 11)		Northeast (*n* = 9)		South (*n* = 15)		West (*n* = 12)		
	*n*	%	*n*	%	*n*	%	*n*	%	*n*	%	p*
Maintenance of eligibility											0.4
Very challenging	15	32%	3	27%	2	22%	5	33%	5	42%	
Somewhat challenging	23	49%	3	27%	6	67%	8	53%	6	50%	
Not challenging	9	19%	5	45%	1	11%	2	13%	1	8%	
Not applicable	0	0%	0	0%	0	0%	0	0%	0	0%	
IT issues – document sharing											>0.9
Very challenging	10	22%	1	9%	2	22%	4	27%	3	27%	
Somewhat challenging	18	39%	5	45%	3	33%	6	40%	4	36%	
Not challenging	16	35%	5	45%	3	33%	5	33%	3	27%	
Not applicable	2	4%	0	0%	1	11%	0	0%	1	9%	
IT issues – HIPAA-specific											0.8
Very challenging	6	13%	0	0%	1	11%	3	20%	2	17%	
Somewhat challenging	12	26%	5	45%	2	22%	3	20%	2	17%	
Not challenging	28	60%	6	55%	6	67%	9	60%	7	58%	
Not applicable	1	2%	0	0%	0	0%	0	0%	1	8%	
Staff turnover											0.06
Very challenging	9	20%	1	9%	4	44%	1	7%	3	27%	
Somewhat challenging	11	24%	4	36%	1	11%	6	40%	0	0%	
Not challenging	20	43%	6	55%	3	33%	7	47%	4	36%	
Not applicable	6	13%	0	0%	1	11%	1	7%	4	36%	
Remote work/telework											0.5
Very challenging	9	19%	0	0%	2	22%	5	33%	2	17%	
Somewhat challenging	24	51%	8	73%	4	44%	5	33%	7	58%	
Not challenging	14	30%	3	27%	3	33%	5	33%	3	25%	
Not applicable	0	0%	0	0%	0	0%	0	0%	0	0%	
Churning on and off ADAP											0.12
Very challenging	5	11%	3	27%	0	0%	0	0%	2	17%	
Somewhat challenging	25	54%	5	45%	5	56%	7	50%	8	67%	
Not challenging	15	33%	3	27%	4	44%	7	50%	1	8%	
Not applicable	1	2%	0	0%	0	0%	0	0%	1	8%	
Churning within ADAP programs											0.8
Very challenging	5	11%	2	18%	1	11%	1	7%	1	8%	
Somewhat challenging	24	52%	6	55%	4	44%	6	46%	8	67%	
Not challenging	15	33%	3	27%	4	44%	6	86%	2	17%	
Not applicable	2	4%	0	0%	0	0%	1	100%	1	8%	

* Fisher’s exact test.

Missing response: Missing responses: IT issues Document Sharing (1), Staff Turnover (1), Churning on and off ADAP (1), Churning within ADAP programs (1).

**Table 2:** Innovations and allowances enacted by state AIDS drug assistance programs during first year of the COVID-19 pandemic, overall and by region, 2020.

Innovations/allowances	Total (*n* = 47)		Region								
			Midwest (*n* = 11)		Northeast (*n* = 9)		South (*n* = 15)		West (*n* = 12)		
	*n*	%	*n*	%	*n*	%	*n*	%	*n*	%	p*
More than 30 days of medications											0.8
Did offer	37	79%	9	82%	7	78%	10	67%	11	92%	
Did not offer	6	13%	1	9%	1	11%	3	20%	1	8%	
Considered/considering	3	6%	1	9%	0	0%	2	13%	0	0%	
Not applicable	1	2%	0	0%	1	11%	0	0%	0	0%	
E-certification for eligibility											0.4
Did offer	29	62%	7	64%	3	33%	10	67%	9	75%	
Did not offer	12	26%	3	27%	2	22%	4	27%	3	25%	
Considered/considering	2	4%	0	0%	2	22%	0	0%	0	0%	
Not applicable	4	9%	1	9%	2	22%	1	7%	0	0%	
Newly started to mail medications											0.05
Did offer	11	23%	2	18%	0	0%	2	13%	7	58%	
Did not offer	9	19%	1	9%	2	22%	4	27%	2	17%	
Not applicable	27	57%	8	73%	7	78%	9	60%	3	25%	

*Fisher’s exact test.

### 3.1. Challenges – eligibility and enrollment

Until recently, ADAPs were required to recertify ADAP eligibility every 6 months, which required collection of income documentation and client signatures. ADAPs reported challenges with enrolling and ensuring eligibility of their clients during the first year of the COVID-19 pandemic. The majority of ADAPs indicated the maintenance of client eligibility was the most challenging issue, with 38 ADAPs (81%) describing it as ‘very’ or ‘somewhat’ challenging (Table 1). However, nearly half of the Midwest jurisdictions found maintaining eligibility to be ‘not challenging’ (45%). One Northeast program described the challenge stating, “Case managers being unable to meet with clients in person to obtain/explain documents. Loss of employer insurance and challenges related to getting unemployment information from clients” (Supplementary Table 1).

Churning refers to the transition of clients on and off RWHAP Part B and ADAP services. Churning on and off was identified as ‘very’ or ‘somewhat’ challenging among 64% of ADAPs, while others rated clients churning within ADAP programs (example: from full pay to/from subsidized insurance) similarly (62%). Churning can be caused by a number of factors, including onerous application and re-determination systems that make it hard to stay enrolled in coverage even when individuals are eligible. Churning on or off ADAP can also be due to changes in life circumstances that affect eligibility for certain programs such as abrupt loss of employer-sponsored insurance necessitating enrollment in ADAP, or a loss of income causing movement out of ADAP and into Medicaid. The majority of programs (60%) found that technical issues that hindered client’s ability to transmit eligibility documentation were ‘very’ or ‘somewhat’ challenging. Additionally, the majority (60%) were not challenged by technical issues related to Health Insurance Portability and Accountability Act (HIPAA).

### 3.2. Challenges – administrative

As public health safety net programs operating within state health departments, RWHAP Part B and ADAPs were impacted by the workforce response to the pandemic. Seven programs (16%) noted a decrease in staffing due to re-allocation as part of the public health response to COVID-19, as well as due to quarantine requirements (4%). Interestingly, twenty (43%) ADAPs found that maintaining adequate staffing was ‘not challenging’ while twenty (43%) found it to be ‘very’ or ‘somewhat’ challenging. Programs found working remotely ‘very’ or ‘somewhat’ challenging (70%), with some programs stressing the difficulty establishing rapport with clients (11%) and the limited interpersonal assistance they could provide (16%). Programs also noted the burden to staff’s mental health (9%), with one stating, “The program was acutely aware of and responsive to behavioral health and trauma informed considerations related to workforce staff and patients” (South program). Lastly, 5 (11%) programs described the operational challenges tied to increased program expenditures related to increased demand for services including food delivery, telehealth, and emergency financial assistance.

### 3.3. Challenges – medical

RWHAP Part B programs described the difficulties providing medical services for clients. Closures of facilities following stay-at-home advisories were noted by 9 programs (20%). The implementation of telehealth platforms enabled practitioners to provide routine care while negating transmission risk, but some programs reported diminished benefits due to poor accessibility of these platforms (16%) and the lack of technology-focused educational resources (13%). One Midwest programs described the challenges: “Telehealth provided an avenue to organizations; however, it presents its own challenges and barriers. The technology is new to most service providers, some of whom did not have the bandwidth to quickly adapt. Also, there are many different platforms, some are prohibitively costly for small sized community-based and AIDS Service Organizations to afford. Thus, most of these providers relied on telephonic contact with clients, which was not ideal.” Following the implementation of telehealth platforms and the re-opening of clinics, some programs noted a delay in laboratory services, with a South program adding “Data is stating [*sic*] to reveal overdue labs now that we are more than 12 months into the pandemic.” Five programs experienced issues related to medication access (11%), with some specifying the delays in mail delivery of medications (4%).

### 3.4. Challenges – ancillary services

In addition to disrupting medical care, the COVID-19 pandemic introduced barriers for clients accessing vital ancillary services. Lack of access to transportation services was noted by 5 programs (11%), particularly for clients in “rural areas” (South program) and because, “clients had fear using public transportation for ANY service appointment” (South program). Lack of access to housing services were described among 9% of program, with a West program reporting that “Early on in the pandemic there were a lot of incidents of homeless RW patients requesting hotels, as to avoid congregate settings, especially when winter hit. These incidents clashed with program rules and limited funding for EFA [Emergency Financial Assistance].” Lastly, two programs reported issues with access to dental services due to clinic closures (4%).

### 3.5. Challenges – policy

The Coronavirus Aid, Relief and Economic Security Act -P.L. 116–136 (CARES Act) provided emergency funding for programs to enable uninterrupted service for their clients (22). While appreciative of the funding, some reported challenges with spending the money (7%), with two programs noting contradictory and time-consuming reporting requirements (4%). One program summarized: “The CARES Act resources were greatly appreciated, needed and well used to improve responsiveness in meeting service needs of People Living with HIV. However, this separate funding stream required additional specific administrative burden at multiple levels, from providers having to code and report on additional service category codes, additional line items to be processed during invoicing and monitoring and added reporting requirements. This higher level of administrative burden was especially challenging considering the COVID-19 context. Yet, the additional resources assisted people with HIV with high needs and were valued at all levels” (South program).

### 3.6. Innovations – eligibility and enrollment

As the challenges of the pandemic grew, programs developed innovative strategies to minimize service gaps and provide flexibility to clients. The majority of ADAPs introduced e-certification (completing certification and re-certification entirely electronically) to facilitate client eligibility (62%) while 12 (26%) elected not to offer this service (Supplementary Table 2). Regionally, offering e-certification was more infrequent among Northeast jurisdictions (33%) compared to the Midwest (82%), South (78%) and West (92%). Some programs streamlined the e-certification process for clients through online document sharing platforms (20%). Furthermore, 36% of programs streamlined enrollment with self-attestation for income and residency status and used verbal or text-based signatures. Nine programs chose to introduce grace periods and waivers (20%) to provide flexibility during enrollment: “ADAP staff would get permission over the phone and assist clients with their online application and provide one month of temporary coverage until the client or case manager could provide all documentation” (Midwest program).

### 3.7. Innovations – administrative

Following the initial stay-at-home advisories, programs adapted their workforce model to primarily telework-based. Four programs highlighted how these changes helped to increase organizational structure (9%), with one stating, “ADAP set up internal secure folder structure for daily operational needs for ADAP staff that became very efficient after initial staff training and use” (South program). Others noted how this structure negated physical paperwork and expedited documentation processing for clients (4%). Five programs found that the new model helped case managers maintain client engagement by increasing outreach (11%) with 3 noting how the changes led to increased check-ins (7%). As the pandemic continued, some programs recognized the emotional trauma to staff and clients, and introduced pandemic stress and trauma programming for staff and clients (4%).

### 3.8. Innovations – medical

In response to the pandemic, programs employed a series of measures to minimize gaps in HIV care. Thirteen programs (29%) highlighted new telehealth platforms for facilitating virtual check-ins, particularly following the success of user training (9%) and for jurisdictions who were developing their platform prior to the pandemic (9%). To increase medication access, the majority of ADAPs allowed clients to obtain more than 30 days of medications (60 or 90 day fills) (79%). Almost all West programs (92%) offered this innovation. While over half of ADAPs already had a mechanism for mailing clients their medications (57%), 11 (23%) began offering this service during the pandemic, particularly in the West (58%, *p* < 0.05). Some programs allowed clients to obtain refills early (11%). One Northeast program summarized their innovations in service delivery: “The [program] have implemented steps to further streamline enrollment, and changes to both pharmacy and primary care formularies allow for extended supplies, early refills, telehealth options, and other methods to assist in minimizing exposure to COVID-19 for participants while allowing for uninterrupted access to care.”

## 4. Discussion

Our study highlights the breadth of challenges to state RWHAP Part B and ADAPs during the initial phases of the COVID-19 pandemic, and the innovations they developed in order to continue providing prescription drug assistance and medical care. For ADAPs, the maintenance of client eligibility was identified as the most challenging issue, and the majority allowed clients to obtain more than 30 days of medication. One-fifth to one-quarter of RWHAP Part B programs noted challenges with clinic closures and delayed lab services, and one-third described the implementation of an innovative telehealth platform. Our analysis reflects that during the first year of the pandemic the majority of programs employed flexible, innovative policies in response to ongoing challenges.

During the initial months of the pandemic, the Health Resources and Services Administration (HRSA) provided guidance to programs that clarified enrollment and service delivery policy and introduced changes as the pandemic progressed. In September 2020, HRSA released comprehensive documents interpreting existing policy requirements (23) in the context of the pandemic, and encouraged programs to exercise flexibility in determining eligibility, promoting remote documentation processes, and recertification (24, 25). By October 2021, HRSA introduced additional flexibilities by eliminating the 6 month recertification requirement and continuing to allow programs to confirm eligibility in accordance with their policies and procedures (26). Further research is warranted to assess how these policies impact client outcomes, and if any can be permanently adopted to optimize care and prevention into the future.

Our findings detailing the program’s perspectives are similar to what has been found for providers of the RWHAP. Kaiser Family Foundation’s survey of directly funded RWHAP medical provider grantees found similar service delivery innovations during the pandemic (12). Nine out of 10 RWHAP clinicians reported offering multi-month ART prescriptions (12) while we found that 8 out of 10 ADAPs allowed ART prescriptions for more than 30 days. Almost 40% of RWHAP clinicians reported a change in payer mix, primarily an increase in clients who were uninsured, and this is in line from a programmatic perspective with more than half of ADAPs reporting that clients churning on and off ADAP was ‘very’ or ‘somewhat’ challenging. In contrast, while 61% of ADAPs reported an increase in total expenditures for over $200 million between 2019 and 2020 (15), we only found 11% of programs noting this trend in the open-ended responses. As RWHAP Part B programs and ADAPs are vital in ensuring continued access to HIV care, the identification and uptake of innovative policies and practices to prevent coverage gaps is critical and will be particularly relevant as Medicaid’s continuous coverage requirement is repealed as the PHE concludes.

Since the completion of our study, the 2023 National RWHAP Part B and ADAP Monitoring Project Report was published and provides additional insight regarding COVID-19 innovations that were implemented in 2020 and 2021, particularly in the digital public health sphere (27). Of the 37 states who utilized e-consent as of 2022, 33 programs (89%) indicated they were ‘somewhat likely’ or ‘very likely’ to continue its use. Regarding the use of secure document sharing for enrollment and recertification, 45 states (98%) were ‘very likely’ to continue, and only one state (2%) indicated they were ‘very unlikely’ to continue. The high level of continuation of these digital public health tools seems to signal that programs found this beneficial. While both of these digital tools have a clear utility and high acceptance among these safety net programs, it is critical to recognize and minimize the barriers to access for communities negatively affected by social determinants of health (28). Safety net programs need to adapt and innovate on digital public health tools so that they adequately serve and address the needs of their key populations.

The strengths of this work include that it is a national sample with a high response rate, and the study was conducted close to the time period in question so recall bias was likely minimal. Furthermore, a study from a national scope has not been published to date. The limitations include the possibility of non-response bias (29) as participating programs may differ from those that did not respond. Additionally, the response rate for the open ended questions was lower than for the quantitative survey questions. Finally, because it was a cross-sectional design, we could not identify dynamic challenges and innovation as the COVID-19 pandemic unfolded. Future work in this area is needed.

Overall, our findings characterize the measures RWHAP Part B programs and ADAPs took to provide clients with essential services during the first year of the COVID-19 pandemic. Other public health programs may learn from these HIV programs and adopt similar innovations, particularly the digital public health tools including secure document sharing via an online platform and e-certification for eligibility. Future studies should evaluate the impact these innovations had for patients, what potential barriers inhibit their widespread use for programs and patients, and what adopted flexibilities can be sustained to optimize service delivery during later phases of the pandemic and post-pandemic.

## Data availability statement

The data analyzed in this study is subject to the following licenses/restrictions: some of the data in this study are publicly available: https://nastad.org/partb-adap-2021-2022-report. Additional data was accessed through a data use agreement with NASTAD. Interested parties can contact NASTAD to discuss data access with a data use agreement. Requests to access these datasets should be directed to https://nastad.org/.

## Author contributions

KM, AmS, AnS, AK, TH, AH, JK-M, contributed to the conception and design of the study. KM, AmS, ZA, AnS, contributed to the acquisition of the data. KM, AnS, ZA, ES, JK-M contributed to the statistical analysis, interpretation, and visualization of data. KM, AnS, AK, AnS, AH, JK-M wrote sections of the manuscript and provided revisions. All authors read and approved the submitted version.

## Funding

This work was supported by the National Institute of Allergy and Infectious Diseases at the National Institutes of Health (NIH) (Grant No. K08AI136644 to KM; Grant No. R01AI170093 to KM). The content is solely the responsibility of the authors and does not necessarily represent the official views of the National Institutes of Health. This work was also supported by the University of Virginia Global Infectious Diseases Institute and the University of Virginia Division of Infectious Diseases and International Health.

## Conflict of interest

KM reports previous stock ownership in Gilead Sciences, Inc. KM also reports unpaid leadership positions: member of the Ryan White Medical Providers Coalition Steering Committee and Chair of the Advisory Committee to Virginia Medication Assistance Program. AK reports being a paid consultant for NASTAD and JSI, working on RWHAP Part B/ADAP technical assistance, and a paid consultant for the HRSA HIV/AIDS Bureau for their Division of State HIV/AIDS Programs. AS reports stock ownership in Merck & Company.

The remaining authors declare that the research was conducted in the absence of any commercial or financial relationships that could be construed as a potential conflict of interest.

## Publisher’s note

All claims expressed in this article are solely those of the authors and do not necessarily represent those of their affiliated organizations, or those of the publisher, the editors and the reviewers. Any product that may be evaluated in this article, or claim that may be made by its manufacturer, is not guaranteed or endorsed by the publisher.
