# Supplementary material for: Ryan White HIV/AIDS Part B and AIDS Drug Assistance Programs during COVID-19: safety net public health programs’ challenges and innovations
Source: Front Public Health. 2023 Jul 31;11:1172009. doi: 10.3389/fpubh.2023.1172009 (PMC10425265; doi:10.3389/fpubh.2023.1172009)
Supplement: Supplementary file 1 [file Table_1.docx]

Supplementary Material

Ryan White HIV/AIDS Part B and AIDS Drug Assistance Programs During COVID-19: Safety Net Public Health Programs’ Challenges and Innovations

# Supplementary Figures and Tables

## Supplementary Table 1

| **Supplementary** **Table 1:** Codes, Representative Quotes, Presence, and Frequency from Analysis of Ryan White HIV/AIDS Part B and AIDS Drug Assistance Program’s Free-Text Responses Describing COVID-19-related Challenges, ADAP Monitoring Report 2021-2022 | | | |
| --- | --- | --- | --- |
| Codes / *Sub-codes* | Representative Quote | Presence (% of responses) | Frequency |
| **Eligibility and enrollment** |  |  |  |
| Churning between and within programs | "The churn in and out of ADAP was challenging in the first half of 2020, and stabilized thereafter." (Northeast program) | 5 (11%) | 6 |
| Technical and administrative issues | "Processing enrollments on time, staffing demands, vacancies/workforce-in COOP mode for a long period, delays from pharmaceuticals processing Rebates and many also had staff changes." (Northeast program) | 3 (7%) | 3 |
| Privacy concerns | "Initiated Docusign to better serve clients in a remote manner. This initiative was not completed due to HIPAA concerns with [West program]. This process is still being worked on for future use." (West program) | 3 (7%) | 4 |
| **Administrative** |  |  |  |
| Lack of interpersonal assistance | "Difficulty in provision of clients services without direct contact; challenged to respond to significant increase in request for emergency financial assistance and to provide PPE to clients" (Midwest program) | 7 (16%) | 7 |
| Difficulty establishing rapport with clients | "Subrecipient agencies report similar difficulties regarding COVID-19, including trying to connect with and establish rapport with newly diagnosed clients over the phone instead of in-person, losing clinic space to COVID-19 testing, and institution-wide pay cuts due to lost revenue." (Midwest program) | 5 (11%) | 7 |
| Decreased outreach capacity | "It also limited some of our outreach capacity while in-person visits were reduced. We tried to increase outreach via virtual visits (telehealth with video and phone outreach), but we did have some clients that were just more difficult to keep engaged, some of whom were limited in their capacity to contact us because they lack stable housing and phone/internet access." (West program) | 2 (4%) | 2 |
| *Operational* |  |  |  |
| Processing time / mailed paperwork delays | "In addition, there were various delays with the US postal delivery system, which created difficulties for some clients in being able to submit required case management enrollment documentation in a timely manner. These delays also created issues with receiving mail order medication, in some cases" (Midwest program) | 9 (20%) | 9 |
| Increased Program expenditures | "ADAP program utilization and expenses remain at a consistently high level, with multi-year projections showing costs that exceed revenue given the declines in Ryan White Part B funding. Enrollment and expenditures have increased due to the COVID-19 emergency." (Northeast program) | 5 (11%) | 5 |
| Project delays | "This disruption, along with canceling of in-person events and adapting to holding meetings and conferences online, significantly slowed several projects, including the medical case management standards revision and the revision of the clinical quality management plan." (Midwest program) | 3 (7%) | 3 |
| *Staffing* |  |  |  |
| Decreased staffing due to re-allocation for COVID-19 | "Healthcare workforce efforts redirected to focus on COVID-19 screenings and treatment." (South program) | 7 (16%) | 7 |
| *Health and safety* |  |  |  |
| Mental health: burnout, stress, trauma, depression | "The program was acutely aware of and responsive to behavioral health and trauma informed considerations related to workforce staff and patients. Department leadership continues to address solutions oriented response to the ongoing impact of pandemic related trauma." (South program) | 4 (9%) | 5 |
| Lack of PPE | "Providers struggled to procure PPE and other hygiene supplies due to supply shortages." (South program) | 3 (7%) | 3 |
| Decreased staffing due to quarantine | "Some provider staff acquired COVID-19 and agencies had to temporarily shut down for deep cleans and staff quarantines/isolations." (South program) | 2 (4%) | 3 |
| **Medical** |  |  |  |
| *Operational* |  |  |  |
| Lab services closed or delayed | "Most service providers temporarily closed or opened for limited services, but then quickly transitioned into telehealth and telemedicine. Data is stating to reveal overdue labs now that we are more than 12 months into the pandemic." (South program) | 10 (22%) | 10 |
| Clinic closures | "Challenges associated with limited access to health care and/or medical case management providers given stay-at-home advisories and temporary closures of medical facilities." (Northeast program) | 9 (20%) | 9 |
| *Telehealth* |  |  |  |
| Telehealth illiteracy and accessibility | "Telehealth provided an avenue to organizations; however, it presents its own challenges and barriers. The technology is new to most service providers, some of whom did not have the bandwidth to quickly adapt. Also, there are many different platforms, some are prohibitively costly for small sized community-based and AIDS Service Organizations to afford. Thus, most of these providers relied on telephonic contact with clients, which was not ideal." (Midwest program) | 7 (16%) | 7 |
| Lack of telehealth resources | "Some agencies needed to purchase equipment and train staff on adopting telemedicine and telehealth, but most were up and quickly running to prevent any gaps in services." (South program) | 6 (13%) | 7 |
| Difficult transition to telehealth | “Most of their challenges were IT related (some agencies did not have access to their client records since they don't have any type of electronic record, needed more laptops and peripherals for people to telework, needed telemed carts and peripherals).” (South program) | 3 (7%) | 3 |
| *Medication Access* |  |  |  |
| Issues with Medication Access | “Delays from pharmaceuticals processing Rebates and many also had staff changes” (Northeast program) | 5 (11%) | 6 |
| Mailed medication delays | "In addition, there were various delays with the US postal delivery system… These delays also created issues with receiving mail order medication, in some cases" (South program) | 2 (4%) | 2 |
| **Ancillary Services** |  |  |  |
| Decreased access to transportation services | "Clients had fear using public transportation for ANY service appointment." (South program) | 5 (11%) | 5 |
| Decreased access to housing services | "Additionally, early on in the pandemic there were a lot of incidents of homeless RW patients requesting hotels, as to avoid congregate settings, especially when winter hit. These incidents clashed with program rules and limited funding for EFA. Some of this has been resolved with the introduction of the vaccine." (West program) | 4 (9%) | 5 |
| Decreased access to dental services | "COVID-19 prevented access to some services such as dental services and lab services, but providers were able to set up virtual/telemedicine visits for clients for other services." (South program) | 2 (4%) | 2 |
| **Policy** |  |  |  |
| Challenges with CARES Act | "The biggest challenges related to COVID-19 were: (1) when rolling out the CARES Act grants, HAB gave contradicting or changing requirements and instructions, making it difficult to plan and prepare, (2) our department decided that we could not augment existing contracts with CARES Act funding; executing new contracts took over five months and meant that providers could not use the funding until the contracts fully executed, (3) our department redirected many staff who would have managed the new CARES Act grants to COVID-19 response, (4) HAB instituted new monthly data reporting for agencies who received CARES Act grants, during a time when many of their staff were being redirect to local COVID-19 responses; even after an agency had spent all their CARES Act funds, HAB expected them to continue reporting under the contract expired." (West program) | 3 (7%) | 4 |
| Reporting requirements | "The CARES Act resources were greatly appreciated, needed and well used to improve responsiveness in meeting service needs of People Living with HIV (PLWH). However, this separate funding stream required additional specific administrative burden at multiple levels, from providers having to code and report on additional service category codes, additional line items to be processed during invoicing and monitoring and added reporting requirements. This higher level of administrative burden was especially challenging considering the COVID-19 context. Yet, the additional resources assisted PLWH with high needs and were valued at all levels." (South program) | 2 (4%) | 2 |
| Abbreviations: PPE - Personal Protective Equipment, COOP – Continuity of Operations, COVID-19 – Coronavirus disease 2019 (COVID-19), PLWH - People Living with HIV, HAB – HIV/AIDS Bureau, CARES Act - Coronavirus Aid, Relief and Economic Security Act -P.L. 116- 136, AIDS Drug Assistance Program (ADAP), PrEP – Pre-exposure Prophylaxis, HIPAA – Health Insurance Portability and Accountability Act | | | |

## Supplementary Table 2

| **Supplementary Table 2:** Codes from Analysis of Ryan White HIV/AIDS Part B and AIDS Drug Assistance Program’s Free-Text Responses Describing COVID-19-related Innovations, ADAP Monitoring Report 2021-2022 | | | |
| --- | --- | --- | --- |
| Codes / *Sub-codes* | Representative Quote | Presence (% of responses) | Frequency |
| **Eligibility and enrollment** |  |  |  |
| Confidential self-attestation, e-consenting, verbal, or text signatures | "[Midwest program] created forms that subrecipient staff could utilize better when doing assessments over the phone while also incorporating new COVID policy changes, which included options for self-attestation of income and residency, as well as the ability to sign electronically." (Midwest program) | 16 (36%) | 18 |
| Introduction of "grace periods" and waivers | "If they requested assistance, ADAP staff would get permission over the phone and assist clients with their online application and provide one month of temporary coverage until the client or case manager could provide all documentation to ADAP." (Midwest program) | 9 (20%) | 10 |
| Secure document sharing and email applications | "Permitted applications to be emailed instead of faxed or mailed." (Northeast program) | 9 (20%) | 9 |
| Allowances of >6 months of continued eligibility | "Changed processes to allow for easier eligibility determination, suspended built-in system audit to allow continued eligibility past the 6-month recertification period." (Northeast program) | 2 (4%) | 2 |
| New short term eligibility | "For any new client, utilizing the Brief Assessment allowed the client to be eligible for services for 45 days." (South program) | 2 (4%) | 2 |
| Waiving Premiums | "Specific COVID-19 State of Emergency policy changes included the pre-payment of health insurance premiums for eligible clients actively enrolled in ADAP" (Northeast program) | 2 (4%) | 2 |
| Maintenance of a mail enrollment option | "Ability to print and mail client approval letters due to working remote. [Midwest program] clients that did not have a Case Manager ensuring their renewal applications were mailed to them in enough of time to avoid gaps in services." (Midwest program) | 2 (4%) | 2 |
| **Administrative** |  |  |  |
| *Operational* |  |  |  |
| Increased client outreach | "ADAP staff called clients on a monthly basis that did not have access to a computer and were on a list to receive paper applications that were due to recertify and/or verify to see if they needed help with their online application." (Midwest program) | 5 (11%) | 5 |
| Increased organization | "ADAP set up internal secure folder structure for daily operational needs for ADAP staff that became very efficient after initial staff training and use." (South program) | 4 (9%) | 2 |
| Increased client check-ins | "A lot of the appointments with clients were over the phone, so some clients fell out of care, however our Case Managers did a great job of staying engaged with the clients." (South program) | 3 (7%) | 3 |
| Increased preparedness for future events | "Transition to telework for ADAP was seamless due to previous emergency preparedness and continuity of operations planning that allowed for rapid implementation" (West program) | 2 (4%) | 2 |
| Decreased need for physical paperwork | "Set up secure e-folders for bilateral document sharing and exchange between ADAP and subrecipients /providers. Required a lot of education and prompting for consistent external use in the beginning and lots of reminders because content clears after 7 days for adherence to best HIPAA practice. Folder structure significantly reduces need for faxing, regardless of teleworking status of ADAP staff." (South program) | 2 (4%) | 2 |
| Pandemic stress / trauma programming | "Acknowledging this reality, [Northeast program] partnered early on with CAI Global to bring a Trauma Informed Care (TIC) lens to wellness and healing. TIC provides skills-based training to assist providers and frontline staff address COVID-related post-traumatic stress and helps both frontline staff and vulnerable populations cope with the psychological and emotional effects of the pandemic." (Northeast program) | 2 (4%) | 2 |
| **Medical** |  |  |  |
| *Medication Access* |  |  |  |
| Early refills | "We approved a series of temporary policy changes that maintain fidelity to legislative obligations while ensuring sustained access to HIV care and treatment during the public health emergency... Specific COVID-19 State of Emergency policy changes included the pre-payment of health insurance premiums for eligible clients actively enrolled in ADAP; reimbursement of pharmacies for prescription medication charges submitted for “early” fills (prescriptions filled before the usual refill approval date), as allowed by the insurer;" (Northeast program) | 5 (11%) | 5 |
| Pharmacy home delivery | "Encouraged point-of-sale pharmacies, at their discretion to deliver medication to clients home if the service is permitted." (Northeast program) | 3 (7%) | 3 |
| >30 day supply | "As a result of the COVID-19 public health emergency, the [Northeast program] have implemented steps to further streamline enrollment, and changes to both pharmacy and primary care formularies allow for extended supplies, early refills, telehealth options, and other methods to assist in minimizing exposure to COVID-19 for participants while allowing for uninterrupted access to care." (Northeast program) | 2 (4%) | 2 |
| *Telehealth* |  |  |  |
| New telehealth platform | "COVID-19 prevented access to some services such as dental services and lab services, but providers were able to set up virtual/telemedicine visits for clients for other services. Access to medications was not impacted." (South program) | 13 (29%) | 14 |
| Ease of transition due to pre-existing platform | "[South program] has been building telehealth capacity in the last few years for the RWHAP B provider network, which really helped us maintain continuity of services throughout the pandemic and during lockdowns." (South program) | 4 (9%) | 6 |
| Provider training and webinars | "[Midwest program] utilized large-group demonstrations, small breakout sessions with individual teams, and a hand-ons practice workbooks to deliver the training." (Midwest program) | 4 (9%) | 4 |
