# Supplementary material for: Ryan White HIV/AIDS Part B and AIDS Drug Assistance Programs during COVID-19: safety net public health programs’ challenges and innovations
Source: Front Public Health. 2023 Jul 31;11:1172009. doi: 10.3389/fpubh.2023.1172009 (PMC10425265; doi:10.3389/fpubh.2023.1172009)
Supplement: Supplementary file 2 [file Data_Sheet_1.DOCX]

# Supplementary Data

## Supplementary Material

Open-ended Questions

1. PART B- Please describe any challenges encountered due to COVID-19 that are relevant to your Part B program in 2020 or 2021.
2. ADAP- Other COVID-related innovations/allowances considered or offered?
3. ADAP- Other COVID-related challenges?

Likert-Style questions

1. COVID-related innovations/allowances
   1. E-certification for eligibility
   2. More than 30 days of meds
   3. Newly started to mail medications to ADAP clients
2. COVID-related challenges
   1. Maintenance of eligibility
   2. Churning within ADAP programs (health insurance plan from/onto Direct ADAP)
   3. Churning on and off ADAP (from/onto employer-based insurance, Medicaid)
   4. Remote work / telework of health department staff
   5. Health department staff turn over
   6. IT / issues with document sharing
   7. IT / HIPAA specific issues
